# Beyond standard therapy: clinical transdiagnostic study evaluating mindfulness-based training as an adjunct to CBT for dissociation

**DOI:** 10.3389/fpsyt.2026.1731654

**Published:** 2026-03-31

**Authors:** O. Fedyk, B. Croissant, C. Schulz, R. Hurlemann

**Affiliations:** 1 Department of Psychiatry and Psychotherapy, School of Medicine and Health Sciences, Carl von Ossietzky University of Oldenburg, Oldenburg, Germany; 2 Karl Jaspers Clinic, Bad Zwischenahn, Germany; 3 Schön Klinik Rendsburg, Rendsburg, Germany; 4 AMEOS Klinikum Osnabrück, Osnabrück, Germany

**Keywords:** cognitive-behavioral therapy, dissociation, mindfulness-based training, personality domains, precision psychiatry, trauma-related disorders

## Abstract

**Background:**

Mindfulness-based interventions show promise for trauma-related conditions, yet controlled trials examining their efficacy for dissociative symptoms remain scarce. This feasibility study evaluated whether Mindfulness-Based Training (MBT), when added to cognitive-behavioral therapy (CBT), reduces dissociative symptoms more effectively than CBT combined with guided physical exercise.

**Methods:**

A controlled pseudo-randomized design was employed with 104 participants assigned to either MBT plus CBT (intervention) or CBT plus physical exercise (active control). Dissociative symptoms were assessed using the Dissociative Experiences Scale (DES-II) at baseline, post-treatment, and 12-week follow-up. Robust mixed-effects linear regression models examined treatment effects and potential moderators including sex and personality domains (Personality Inventory for DSM-5 - PID-5).

**Results:**

MBT did not outperform the active control condition. Both groups showed modest improvements in dissociation scores. Exploratory analyses identified sex and antagonism as potential moderators of treatment response.

**Conclusions:**

MBT as an adjunct to CBT did not demonstrate superior efficacy compared to CBT plus guided physical exercise for reducing dissociative symptoms. Both groups improved modestly during treatment. Moderation findings regarding sex and personality traits are exploratory, not pre-registered, and require independent replication. The study supports the feasibility of integrating low-intensity MBT into clinical settings rather than establishing its efficacy.

## 1 Introduction

Neurobiological research indicates that trauma spectrum disorders are associated with reduced hippocampal and amygdala volume, along with impaired ventromedial prefrontal cortex function (1–3). On the other hand, functional MRI (fMRI) studies suggest that meditation practice increases connectivity between the amygdala and medial prefrontal cortex, making mindfulness a potentially suitable approach for trauma survivors suffering from dissociative symptoms (4, 5).

Despite growing interest in mindfulness-based interventions for trauma-related conditions, high-quality controlled trials evaluating their efficacy for dissociative phenomena remain limited (6–9). Research limitations include small sample sizes, lack of standardization, and short follow-up periods (10). Mindfulness training may help alleviate symptoms of detachment and disconnection by enhancing present-moment awareness and grounding (11, 12).

Mindfulness-based interventions offer practical advantages: they are cost-effective, can be combined with existing therapies, and provide patients with self-management tools. Unfortunately, many studies so far have focused on intensive programs like Mindfulness-Based Stress Reduction (MBSR), which can be difficult to implement in routine clinical settings (13).

This feasibility study evaluated the effectiveness of a low-intensity, standardized mindfulness-based group training program designed for clinical population. The primary objective was to determine whether dissociative symptom reduction in the intervention group significantly exceeds that of treatment as usual (TAU). Secondary objectives included examining whether personality domains measured by Personality Inventory for DSM-5 (PID-5) modulate treatment response and identifying potential prognostic factors.

## 2 Methods

### 2.1 Participants

Participants were recruited from three psychotherapeutic wards at a psychiatric hospital in Osnabrück, Germany. Sample size calculations based on previous research (14) indicated that 52 participants per group were required to detect a medium effect size (d = 0.52) with 80% power and α = 0.05.

**Figure 1:**
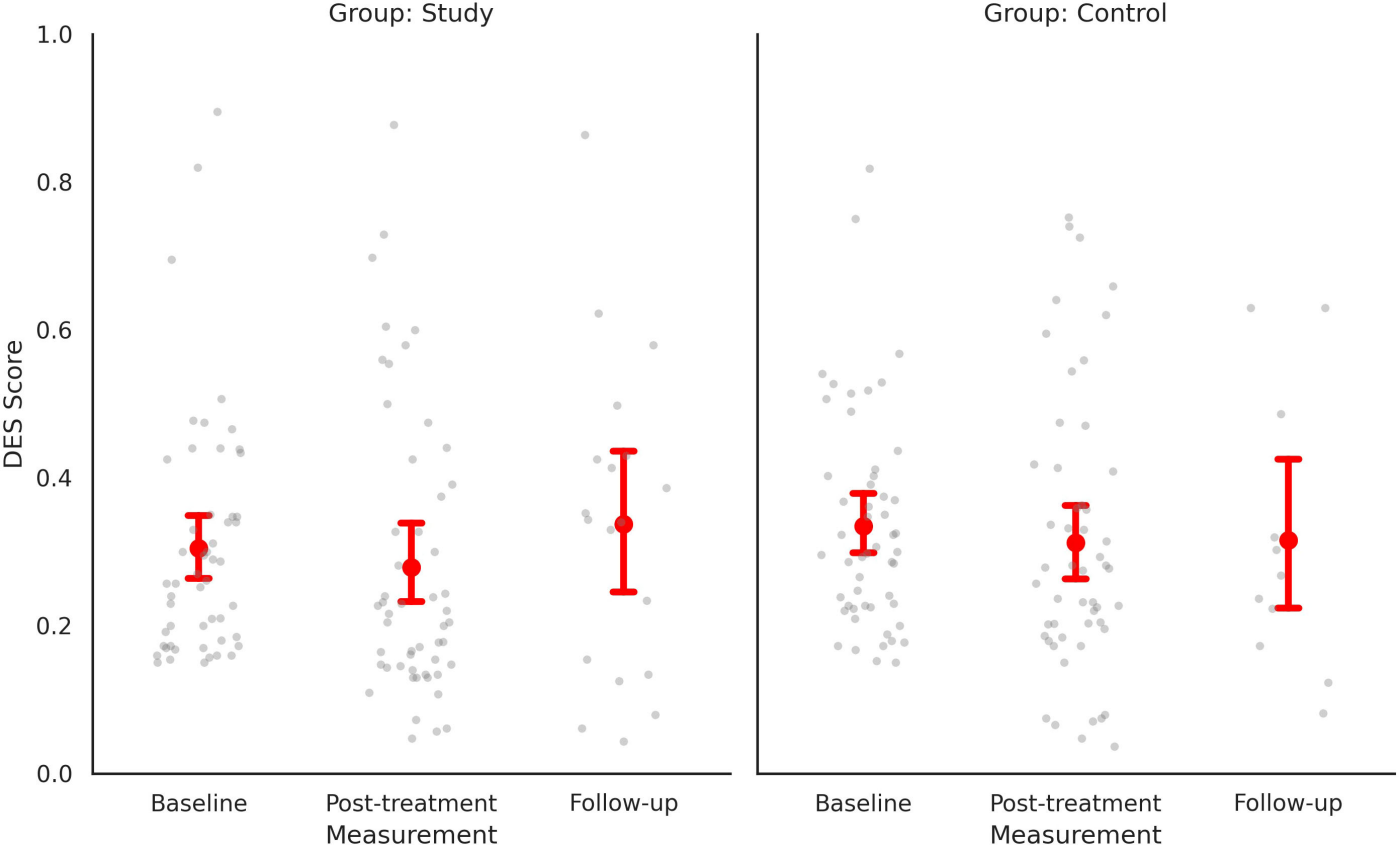
CONSORT 2025 flow diagram. Participant flow through screening, allocation, follow-up, and analysis. Analyzed for primary outcome: n = 52 per group; reasons for discontinuation and loss to follow-up are shown for each arm.

Of 205 patients screened, 104 met inclusion criteria (79 female, 25 males, see CONSORT Flowchart - **Figure 1**). Participants were aged 18–65 years and scored ≥15 on the DES-II. Exclusion criteria included severe psychiatric disorders (psychosis, dementia), acute suicidality, drug dependence, and pregnancy. The largest diagnostic subgroups were major depressive disorder (MDD, n = 50), posttraumatic stress disorder (PTSD, n = 48), and personality disorders (n = 33, including 28 with borderline personality disorder) basic descriptive statistics are presented in **Table 1**, disaggregated by group in the results section, **Table 2** A detailed list of primary and comorbid diagnoses is provided, **Tables 3**, **4**.

**Table 1:** Descriptive statistics.

Variable	Minimum	Maximum	Mean	SD	Q1	Q3
Age	18	65	36.01	12.52	25.00	47.00
DESII Pre	0.150	0.896	0.31974	0.155	0.200	0.399
CAMS-R Pre	12	40	22.47	4.787	19.00	25.00
Isolation	0.450	2.640	1.627	0.526	1.225	2.030
Psychoticism	0.230	2.870	1.374	0.591	0.930	1.830
Disinhibition	0.430	2.590	1.529	0.470	1.163	1.815
Negative Affect	0.240	3.000	1.929	0.556	1.560	2.290
Antagonism	0.000	2.680	0.559	0.531	0.137	0.853

**Table 2:** Demographic characteristics by group.

Measure	Control	Study
n	52	52
Age, years, M (SD)	34.7 (12.1)	37.3 (12.9)
Female, n (%)	37 (71.2%)	42 (80.8%)
Male, n (%)	15 (28.8%)	10 (19.2%)

Age is reported as mean with standard deviation in parentheses.

**Table 3:** Diagnosis contingency tables.

Group S/C			
ICD10 Diagnosis	Control	Study	Total
F3x	13	17	30
F4x	26	25	51
F5x	2	6	8
F6x	11	4	15
Total	52	52	104

**Table 4:** Collapsed ICD-10 main diagnostic categories and comorbidity by group.

Measure	Control, n (%)	Study, n (%)
Main diagnosis (ICD-10, collapsed)		
Mood (affective) disorders (F30–F39)	14 (26.9%)	17 (32.7%)
Neurotic, stress-related and somatoform disorders (F40–F48)	25 (48.1%)	25 (48.1%)
Behavioral syndromes associated with physiological disturbances (F50–F59)	2 (3.8%)	6 (11.5%)
Disorders of adult personality and behavior (F60–F69)	11 (21.2%)	4 (7.7%)
Comorbidity (additional diagnosis fields)		
Any comorbid diagnosis	43 (82.7%)	45 (86.5%)
0 additional diagnoses	9 (17.3%)	7 (13.5%)
1 additional diagnosis	19 (36.5%)	22 (42.3%)
2 additional diagnoses	24 (46.2%)	23 (44.2%)

Diagnostic categories are based on ICD-10 F-chapter groupings collapsed from the primary diagnosis field.

### 2.2 Study design

Participants were allocated to groups based on scheduling availability, independent of study staff. While not strictly randomized, allocation can be considered pseudo-random as neither patients nor investigators influenced assignment. Upon enrollment, each participant was assigned a numerical identification code to ensure data confidentiality. Thus, blinding was implemented only during the data analysis phase, when group assignments were concealed from the analysts.

The intervention group received Mindfulness-based training (MBT) in addition to standard cognitive behavioral therapy (CBT). The control group received CBT plus weekly outdoor exercise (endurance training, supervised walking) of comparable duration, but no mindfulness exercises, to control for non-specific therapeutic effects. Both groups received symptomatic pharmacological treatment when clinically indicated. CBT included the identification and modification of dysfunctional thoughts, behavioral activation, coping skills training, homework assignments supporting behavioral and cognitive change, and participation in psychoeducational groups aimed at enhancing self-awareness and emotional regulation; but also forms of therapy specific to certain disorders, such as expositions or Eye Movement Desensitization and Reprocessing (EMDR) therapy in the case of PTSD.

The MBT protocol was adapted from the manual by Beckmann and Wolf-Arehult (15) and consisted of eight weekly 60-minute group sessions. MBT was delivered in a dedicated group room on the ward with a calm, low-stimulation environment, and sessions were facilitated by a trained clinician. Sessions included body scan exercises, guided visualizations, group reflection, and assigned free time practice.

### 2.3 Measures

The primary outcome was the Dissociative Experiences Scale (DES-II), measuring four dimensions: derealization/depersonalization, conversion, absorption, and amnesia (16, German version: 17) at three timepoints: pre, post and follow up. As a basic fidelity check was implemented the one-dimensional, 12-Item Cognitive and Affective Mindfulness Scale Revised (CAMS-R, 18). Personality traits were measured with the Personality Inventory for DSM-5 (PID-5), evaluating Negative Affect, Detachment, Antagonism, Disinhibition, and Psychoticism (19).

### 2.4 Statistical analysis

Analyses were conducted using R (version 4.3.1). Robust linear mixed-effects models (RLMER) with M-estimation were applied to account for outliers and non-normality (20, 21). The DES-II score was log-transformed to reduce heteroscedasticity and stabilize variance. Random effects for participant identifiers modeled intra-subject correlations. Predictors included treatment group, time, sex, age, and PID-5 domains, with interaction terms to explore differential treatment effects.

### 2.5 Protocol deviations

The preregistered protocol (DRKS00026604) originally planned to compare once-weekly versus three-times-weekly mindfulness sessions. Due to practical constraints, only the once-weekly format was implemented.

### 2.6 Drop out and missing data

A screening and dropout log identified reasons for non-participation or attrition. Qualitative analysis showed that “other/unspecified reasons” (44.8%) and “early discharge or insufficient stay duration” (20.7%) were the primary drivers. Additional factors included questionnaire non-completion (10.3%), concurrent study participation (6.9%), and substance use (6.9%). These results suggest that attrition in this naturalistic inpatient setting was primarily influenced by organizational constraints and patient-related factors such as questionnaire burden.

Missing data predominantly affected post-treatment and follow-up measures (DES-II, CAMS-R) due to discharge or loss of contact. To address this, we utilized robust mixed-effects models to include all available observations without imputation.

## 3 Results

### 3.1 Baseline characteristics

Most variables did not meet normality assumptions (Shapiro-Wilk test). Mann-Whitney U tests revealed no significant baseline differences between groups on demographic or clinical variables (see **Table 5**). Chi-square tests showed no significant differences in DES-II scores by sex (p = 0.251,**Table 6**) or collapsed diagnosis (p = 0.121, **Table 7**). Spearman correlations confirmed expected associations between baseline dissociation and personality domains: Isolation (ρ = 0.466, p < 0.001), Psychoticism (ρ = 0.596, p < 0.001), and Disinhibition (ρ = 0.215, p = 0.029, **Table 8**). The contigency of patients by sex was comparable in both groups, compare **Table 9**. **Table 10** presents the raw scores of the DES-II and its subscales by group at baseline and after the intervention.

**Table 5:** Independent samples MannWhitney U test.

Variable	U	df	p
Isolation	135.000		0.555
Psychoticism	141.500		0.678
Disinhibition	154.000		0.936
Negative Afffect	191.000		0.376
Antagonism	164.000		0.872
Age	103.000		0.147

**Table 6:** Sex: chi-squared tests.

Test	Value	df	p
X²	1.316	1	0.251
N	104		

**Table 7:** Diagnosis chi-squared tests.

Test	Value	Df	p
X²	5.820	3	0.121
N	104		

**Table 8:** Spearman's correlations at baseline.

Variable 1		Variable 2	Spearman's rho		P
DES Pre	–	CAMS-R Pre	-0.091		0.358
DES Pre	–	Age	0.002		0.983
DES Pre	–	Isolation	0.466	***	**< .001**
DES Pre	–	Psychoticism	0.596	***	**< .001**
DES Pre	–	Disinhibition	0.215	*	**0.029**
DES Pre	–	Negative Afffect	0.183		0.063
DES Pre	–	Antagonism	-0.014		0.890

* p < .05, ** p < .01, *** p < .001, Bonferroni-Holm-Adjusted. statistically significant results (p < 0.05) are marked in bold.

**Table 9:** Sex - contingency tables.

Group S/C	Sex		Total
	Male	Female	
Control	15	37	52
Study	10	42	52
Total	25	79	104

**Table 10:** DES-II total score and subscales (Pre–Post) by group.

Scale	Group	Pre n	Pre M (SD)	Post n	Post M (SD)	Change n	Change M (SD)
Amnesia	Control	52	26.92 (18.00)	52	20.41 (18.87)	52	-6.51 (18.00)
Amnesia	Study	52	20.78 (19.45)	52	16.62 (17.12)	52	-4.17 (15.51)
Absorption	Control	43	46.49 (19.32)	46	39.12 (20.01)	38	-3.68 (18.84)
Absorption	Study	45	40.69 (18.24)	43	38.35 (20.89)	36	0.18 (17.08)
Derealization/Depersonalization	Control	43	32.33 (21.75)	46	27.69 (21.42)	38	-2.54 (14.72)
Derealization/Depersonalization	Study	45	27.83 (22.06)	43	25.85 (23.75)	36	2.53 (16.70)
Conversion	Control	42	28.39 (16.18)	45	21.38 (17.85)	37	-5.25 (15.06)
Conversion	Study	41	27.07 (20.44)	35	27.62 (23.05)	27	-1.48 (15.59)
DES-II Total (0–1 scale in file)	Control	52	0.33 (0.15)	52	0.31 (0.19)	52	-0.02 (0.14)
DES-II Total (0–1 scale in file)	Study	52	0.30 (0.16)	52	0.28 (0.19)	52	-0.03 (0.15)

Change = Post − Pre. Sample sizes vary across subscales due to missing data. In the provided dataset, DES-II total scores are stored on a 0–1 scale.

### 3.2 Primary outcome

The primary analysis revealed no significant group × time interaction (β = -0.05, SE = 0.10, 95% CI: -0.25 to 0.14, p = 0.588, **Table 11**). MBT added to CBT did not significantly outperform CBT plus guided physical exercise in reducing DES-II scores. Model fit was adequate (residual quartiles: Q1 = -0.48, Median = -0.03, Q3 = 0.51). Fixed effects explained 44% of variance (marginal R² = 0.44); including random effects increased this to 66% (conditional R²). The ICC of 0.39 indicated substantial individual variability in treatment response.

**Table 11:** Regression coefficients of the fitted RLMER model with DES-II score outcome.

Predictors	Log (DES score)			
	β	SE	CI 95%	P
(Intercept)	-0.98	0.12	-1.23 – -0.74	**< 0.001**
group [study]	0.02	0.10	-0.17 – 0.20	0.852
time [post-treatment]	-0.08	0.07	-0.23 – 0.06	0.253
sex [male]	-0.09	0.13	-0.34 – 0.16	0.475
Age *(centered at M = 36,0 yrs.)*	-0.01	0.00	-0.01 – -0.00	0.018
Isolation *(centered at M = 1.63 score)*	0.28	0.08	0.12 – 0.43	< 0.001
Psychoticism *(centered at M = 1.37 score)*	0.42	0.08	0.27 – 0.57	< 0.001
Disinhibition *(centered at M = 1.53 score)*	0.02	0.09	-0.15 – 0.20	0.781
Negative affect *(centered at M = 1.93 score)*	0.01	0.08	-0.14 – 0.16	0.912
Antagonism *(centered at M = 0.56 score)*	-0.12	0.08	-0.27 – 0.03	0.104
group [study] × time [post-treatment]	-0.05	0.10	-0.25 – 0.14	0.588
group [study] × sex [male]	0.13	0.19	-0.25 – 0.51	0.514
time [post-treatment] × sex [male]	-0.09	0.14	-0.36 – 0.18	0.512
(group [study] × time [post-treatment]) ×sex [male]	-0.07	0.21	-0.48 – 0.34	0.73

β, regression coefficient; *DES-II*, Dissociative Experiences Scale; *RLMER*, Robust linear mixed-effects model; *SE*, standard error; *CI 95%*, confidence interval 95%; *p*, p-value of statistical test. statistically significant results (p < 0.05) are marked in bold.

### 3.3 Exploratory moderator analyses

Estimated marginal means for the model separated into three categorical variables are presented in **Table 12**. They served as the basis for further analysis of contrasts. *Post hoc* contrast analyses (exploratory, not pre-registered) suggested potential sex differences (**Tables 13**, ** 14**). Females in the MBT group showed a modest reduction in DES-II scores (d = 0.45, p = 0.045), while males showed a larger effect (d = 0.97, p = 0.034).

**Table 12:** Estimated marginal means of DES-II scores (back transformed from logarithm scale) across time, group and sex factors adjusted for age and psychological covariates.

Time	Group	Sex	Response	SE	CI 95%
Baseline	control	female	0.29	0.02	0.25 – 0.33
post-treatment	control	female	0.27	0.02	0.23 – 0.30
Baseline	Study	female	0.29	0.02	0.26 – 0.33
post-treatment	Study	female	0.26	0.02	0.23 – 0.29
Baseline	control	male	0.26	0.03	0.21– 0.33
post-treatment	control	male	0.22	0.02	0.18 – 0.27
Baseline	Study	male	0.31	0.04	0.23 – 0.40
post-treatment	Study	male	0.23	0.03	0.17 – 0.29

*SE*, standard error; *CI 95%*, confidence interval 95%; *DES-II*, Dissociative Experiences Scale.

**Table 13:** Regression coefficients of the RLM model n_obs_ = 104.

Predictors	ΔDES _baseline – post-treatment_			
	β	SE	CI 95%	P
(Intercept)	0.02	0.02	-0.02 – 0.06	0.307
Group [study]	0.02	0.03	-0.04 – 0.07	0.563
Sex [male]	4.84 × 10^-3^	0.03	-0.06 – 0.07	0.875
Age *(centered at M = 36,0 yrs.)*	1.50 × 10^-3^	1.11 × 10^-3^	-0.70 × 10^-3^– 3.70 × 10^-3^	0.179
Isolation *(centered at M = 1.63 score)*	-0.02	0.03	-0.08 – 0.03	0.415
Psychoticism *(centered at M = 1.37 score)*	6.86 × 10^-4^	0.03	-0.06 – 0.06	0.981
Disinhibition *(centered at M = 1.53 score)*	-0.03	0.03	-0.09 – 0.04	0.398
Negative affect *(centered at M = 1.93 score)*	-0.03	0.03	-0.08 – 0.02	0.275
Antagonism *(centered at M = 0.56 score)*	0.06	0.03	1.60 × 10^-3^– 0.11	**0.044**

β , regression coefficient; *DES-II*, Dissociative Experiences Scale; *RLM*, Robust linear model; *SE*, standard error; *CI 95%*, confidence interval 95%; *p*, p-value of statistical test; *n_obs_*, number of observations. Statistically significant results (p < 0.05) are marked in bold.

**Table 14:** Results of contrast analysis of DES-II score within time across group and sex.

Contrast	Group	Sex	Est.	SE	Z	P	D	CI 95%
baseline/post-treatment	control	female	1.09	0.08	1.14	0.253	0.27	-0.20 – 0.74
baseline/post-treatment	Study	female	1.15	0.08	2.01	0.045	0.45	0.00 – 0.90
baseline/post-treatment	control	male	1.19	0.14	1.50	0.133	0.56	-0.18 – 1.31
baseline/post-treatment	study	male	1.35	0.19	2.12	0.034	0.97	0.05 – 1.90

*DES-II*, Dissociative Experiences Scale; *SE*, standard error; *z*, statistic of Wald z test; *p*, p-value of Wald z test; *d*, Cohens d effect size; *CI 95%*, confidence interval of effect size.

Age was associated with lower DES-II scores (β = -0.01, p = 0.018). Higher Isolation (β = 0.28, p < 0.001) and Psychoticism (β = 0.42, p < 0.001) predicted higher dissociation scores (**Table 11**).

### 3.4 Follow-up

31 participants completed the 12-week follow-up. Wilcoxon signed-rank tests showed no statistically significant changes from baseline to follow-up in either group (**Table 15**).

**Table 15:** Paired samples Wilcoxon signed-rank test – follow up.

Measure 1		Measure 2	W	Z	df	P
DES S Pre	–	DES S FU	73.500	-0.523		0.707
DES C Pre	–	DES C FU	48.000	1.334		0.099

**Figure 2:**
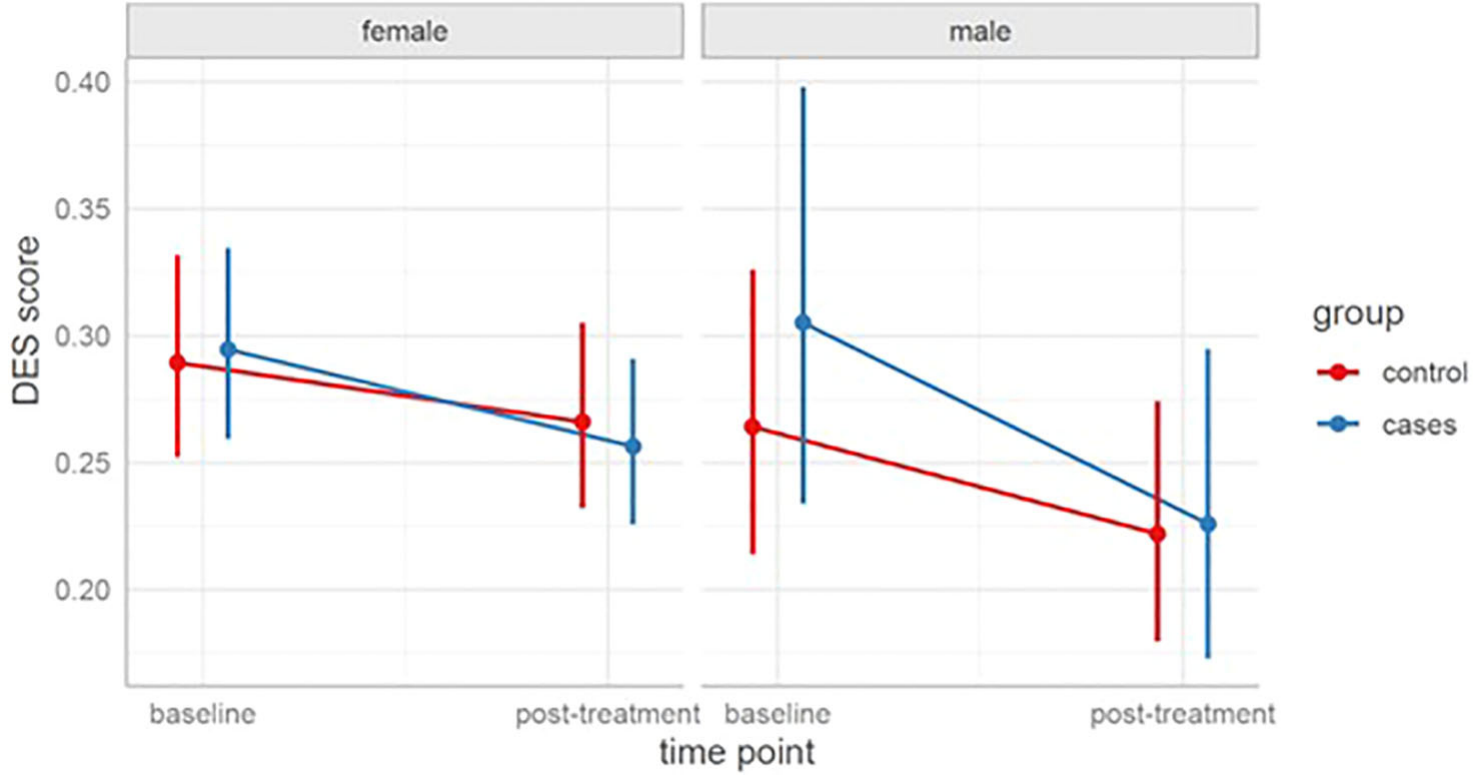
DES-II scores over time by group. Individual participant scores (gray points) and group means with 95% CIs (red points and error bars) are shown for the Study and Control groups at Baseline, Post-treatment, and Follow-up. DES-II total score is scaled from 0 to 1 (higher scores indicate greater dissociative symptom severity). CI, Confidence Interval; DES-II, Dissociative Experiences Scale.

**Figure 3:**
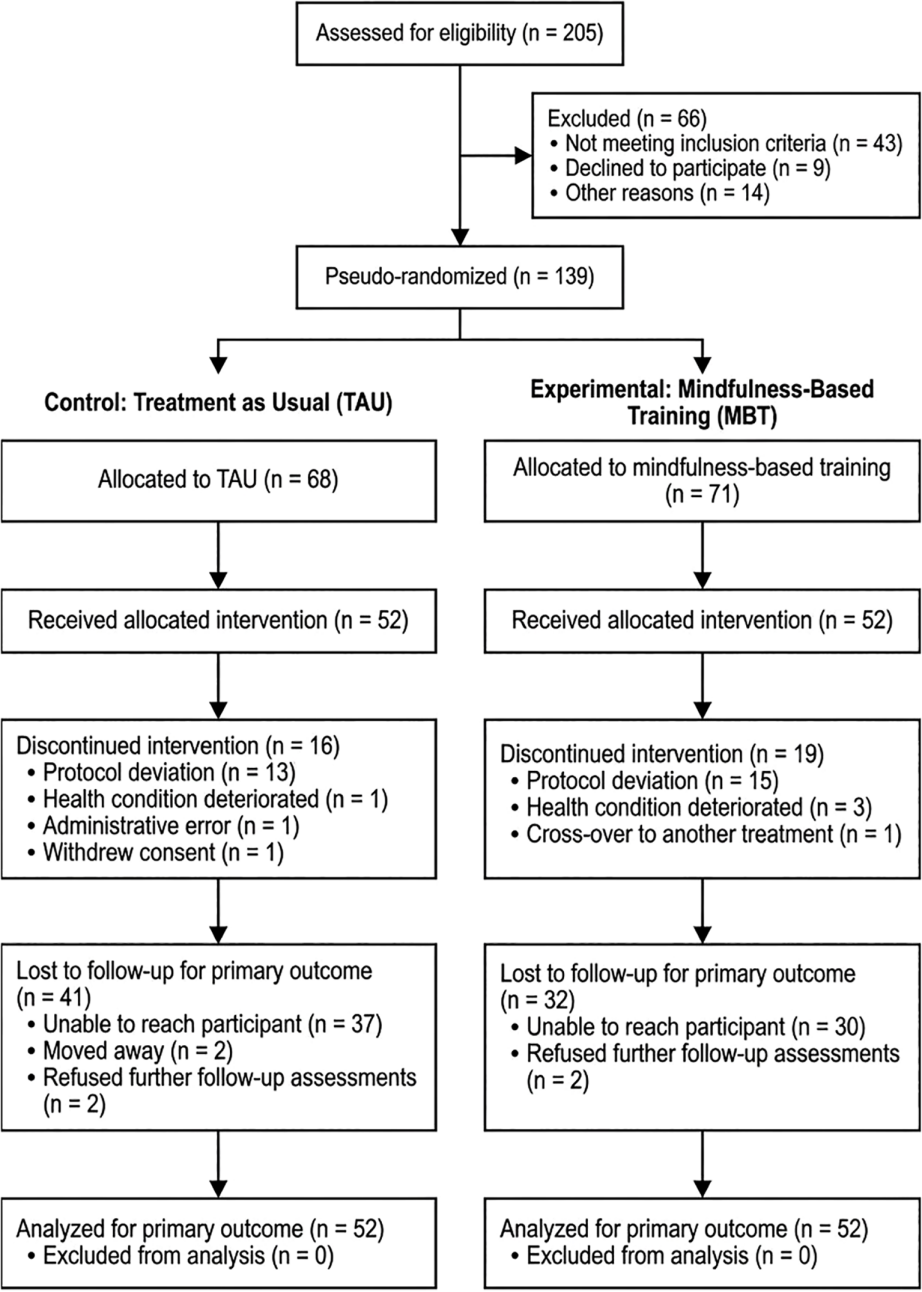
Estimated marginal means of DES-II by time, group, and sex (adjusted). Adjusted estimated marginal means with 95% CIs are shown for females (left) and males (right) at Baseline and Post-treatment for Control and Study groups. Estimates were obtained from a RLMER adjusting for age and psychological covariates. CI, Confidence Interval; DES-II, Dissociative Experiences Scale; RLMER, Robust linear mixed-effects model.

**Figure 2** shows the change in DES-II scores ​over time for both groups at the three measurement time points. **Figure 3** shows these changes in a graph disaggregated by group and gender.

## 4 Discussion

### 4.1 Main findings

This feasibility study found no evidence that adding MBT to CBT produces superior outcomes compared to CBT plus guided physical exercise for treating dissociative symptoms. Both groups showed modest improvements during treatment, but no significant between-group differences emerged. For this reason, the first research hypothesis should be rejected.

The absence of differential effects may reflect several factors. First, both interventions share mechanisms that reduce stress: mindfulness training and physical activity both regulate the autonomic nervous system and may enhance neuroplasticity (22, 23). Second, non-specific therapeutic factors - including the structured hospital environment, social support, and the therapeutic relationship - likely contributed to improvements in both groups.

The increase in mindfulness scores in both groups supports this interpretation, as psychotherapy inherently involves metacognitive elements that may overlap with mindfulness practices.

### 4.2 Exploratory findings

Exploratory analyses identified sex and antagonism as potential moderators of treatment response.

Higher antagonism scores were associated with a greater reduction in dissociative symptoms, a finding that contrasts with typical links between antagonistic traits and emotional detachment (24, 25). However, this association should be interpreted with caution due to low average antagonism levels in the sample and potential regression to the mean.

Psychoticism significantly predicted baseline DES-II scores, indicating that higher levels of eccentricity and perceptual dysregulation are markers for greater dissociative burden at intake. This finding suggests that dissociation is closely linked to personality structure, particularly traits related to cognitive dysregulation. Consequently, assessing these personality domains may provide important context for understanding the severity of dissociative symptoms.

These findings are hypothesis-generating and require replication in adequately powered studies. The small subgroup sizes preclude strong conclusions about differential treatment effects.

### 4.3 Limitations

Several limitations warrant consideration when interpreting the present findings.

First, the pseudo-randomized design, while pragmatic for a naturalistic setting, may introduce selection bias compared to a strictly randomized controlled trial. Second, although the sample size was sufficient for the primary analysis, it limited the statistical power to detect small effects and robustly explore moderators. In this context, the moderation findings must be viewed as exploratory and hypothesis-generating. Third, substantial attrition at the 12-week follow-up (approximately 70% loss) prevented a reliable assessment of the long-term stability of the treatment effects. Fourth, the deviation from the originally preregistered protocol regarding session frequency and sample size may affect the generalizability and comparability of these results.

Furthermore, certain demographic and socioeconomic variables—including formal education level, socioeconomic status (SES), and detailed history of prior psychological treatments — were not systematically recorded. While the clinical setting aimed for a representative inpatient sample, we cannot fully rule out the influence of these unmeasured baseline characteristics on treatment outcomes. Similarly, while medication was administered according to clinical guidelines and monitored by staff, specific data on medication classes and dosages were not included in the analysis, representing a potential unmeasured moderator of efficacy.

Regarding the intervention, we did not formally restrict or monitor participants’ exposure to other mindfulness or relaxation techniques outside the structured sessions. Given the modern clinical environment and access to informal resources, the possibility of “mindfulness leakage” or spontaneous practice within the TAU group cannot be entirely excluded. Despite these limitations, the use of a standardized primary outcome measure (DES-II) and a control group from the same clinical environment provide a robust basis for this initial evaluation of the protocol.

### 4.4 Implications

The study demonstrates that low-intensity MBT can be feasibly integrated into psychiatric inpatient settings. Perhaps, mindfulness training could be successfully applied to those patients who, due to limited mobility or other health contraindications, cannot participate in physical activities. However, the current findings do not support MBT as superior to active control conditions for dissociative symptoms. Future research should employ more refined control conditions and larger samples to better isolate mindfulness-specific effects.

## 5 Conclusions

MBT as an adjunct to CBT did not outperform CBT plus guided physical exercise in reducing dissociative symptoms. Both groups showed modest improvements during treatment. Exploratory moderation analyses suggest potential sex and personality differences in treatment response, but these findings require independent replication. This study supports the feasibility of integrating low-intensity MBT into clinical practice but does not establish its efficacy over active comparison conditions.

## Data Availability

The raw data supporting the conclusions of this article will be made available by the authors, without undue reservation.
